# Immune response, clinical outcome and safety of dendritic cell vaccine in combination with cytokine-induced killer cell therapy in cancer patients

**DOI:** 10.3892/ol.2013.1376

**Published:** 2013-06-04

**Authors:** YU CUI, XUEJING YANG, WEI ZHU, JIALI LI, XIAOJING WU, YAN PANG

**Affiliations:** 1 Department of Oncology, Tianjin Union Medicine Centre, Tianjin 300121, P.R. China;; 2 Shanghai Claison Biotechnology Co., Ltd., Shanghai 201201;; 3 Institute of Oncology, Tianjin Union Medicine Centre, Tianjin 300121, P.R. China

**Keywords:** dendritic cell vaccine, cytokine-induced killer, cancer

## Abstract

The aim of the present study was to determine the clinical value of autologous immunocyte therapy as a standard treatment regimen for patients with cancer. A total of 121 patients with cancer were included in this study. Subsequent to performing leukapheresis using the Fresenius Kabi System, 1×10^7^ dendritic cells (DCs) for the vaccine and 1×10^9^ cytokine-induced killer (CIK) cells for injection were prepared. An analysis of the immune phenotypes of HLA2DR, CD80 and CD83 for the DCs and of CD3, CD8 and CD56 for the CIK cells, as well as negative detection of bacteria and endotoxin, were used as the quality standards. The delayed-type hyper-sensitivity (DTH) skin test was used to measure the immune response, while physical strength, appetite and sleeping status were analyzed for the clinical outcome. Fever, insomnia, anorexia, joint soreness and skin rashes were recorded as side-effects. Patients received the DC vaccination once a week for six weeks and a CIK cell injection six times within four days. In total, 121 cancer patients with primary tumors located in the colorectum (43.0%), lung (15.7%), breast (11.6%), kidney (5.8%), stomach (4.1%) and other regions (19.8%) were included in the study. A positive cell-mediated cytotoxicity response rate of 76.9% was detected by the DTH skin tests. Improvements in physical strength, appetite and sleeping status were observed in 94.1, 83.9 and 76.3% of cases, respectively. None of the serious adverse side-effects that commonly occur during chemotherapy and radiotherapy were observed. During therapy, 69 cases developed a fever that was resolved with antipyretics, dexamethasone or physical cooling, while 28 cases developed insomnia combined with excitement, 19 cases complained of anorexia, 11 cases complained of joint soreness, which was alleviated using analgesics, and 8 cases developed skin rashes. The combined use of CIK cells with a DC-based cancer vaccination strategy may be used to target innate and adaptive immune mechanisms and synergistically promote positive clinical outcomes. The therapy was safe and no serious adverse side-effects similar to those caused by chemotherapy and radiotherapy were observed. The regimen may have a beneficial effect in the future treatment of patients with cancer.

## Introduction

Cancer represents one of the major causes of mortality worldwide (1). More than half of patients suffering from cancer succumb to their condition (1). At present, the major approaches to treating cancer are surgical resection followed by radiation therapy and chemotherapy. These treatments have resulted in significant benefits to patients with the majority of tumor types, and the clinical outcomes have become more satisfactory (2–4). To further increase this trend of improving treatment outcomes, new treatments are necessary; one option proposed for this is immunotherapy (5). It is recognized that multidisciplinary treatments should be used in cancer treatment. The combination of the traditional methods of surgery, chemotherapy and radiotherapy with immunotherapy is a new way for anti-cancer therapies to reduce the mortality of cancer patients. Cancer immunotherapy is a promising cancer treatment method. The dysfunction of the antigen-specific T cells required to kill the cancer leads to cancer cells being able to grow in cancer patients. Active and adoptive T cell immunotherapies generate T cells that may be able to target cancer cells (6,7).

Dendritic cells (DCs) are immune cells that function as antigen-presenting cells. They are able to activate naive CD4^+^ T helper cells and unprimed CD8^+^ cytotoxic T lymphocytes. Active immunotherapy, represented by DC-based regimens, has been used to produce tumor-specific antigen-presenting cells and to generate cytotoxic T lymphocyte responses against cancer cells (8–12). Adoptive immunotherapy, a personalized therapy that uses a patient’s own anti-tumor immune cells to kill cancer cells, may be used to treat several types of cancer, representing a potential therapeutic approach against cancer. The adoptive immunotherapy approach is one of the most effective methods for using the body’s immune system to treat cancer (13). Cytokine-induced killer (CIK) cells are considered to be a heterogeneous population containing the T cell marker CD3 and the natural killer (NK) cell marker CD56. As an adoptive T cell immunotherapy, CIK cells have shown significant cytotoxic activity in clinical studies (14–16).

The purpose of the present study was to determine the cellular immune response in terms of the delayed-type hyper-sensitivity (DTH) skin test and evaluate the subjective clinical outcome and safety of the regimen in cancer patients receiving a DC vaccine in combination with CIK therapy.

## Patients and methods

### Study design

The study was an open-label, single-institution, non-randomized exploratory study performed at the Department of Oncology, Tianjin Union Medicine Center, Tianjin, China. All cases were referred to the Department in August 2012. The study protocol was approved by the hospital’s Ethics Committees and was in accordance with the ‘Treatment with Autologous Immune Cells (T cells and NK cells)’ class III medical techniques policy of the Ministry of Health of China. All patients provided written informed consent prior to treatment.

### Study procedures

Patients with histologically proven cancer or those who had been diagnosed by imaging and serum tumor markers with histological types that could be defined were selected to participate in the present study. Patients were excluded if they had severe renal or coagulation dysfunction or if the total number of peripheral lymphocytes and monocytes was <1×10^9^/l. Subsequent to performing leukapheresis using a Fresenius Kabi System with an ECG monitoring system, 1×10^7^ DCs for the vaccine and 1×10^9^ CIK cells for injection were prepared. An analysis of the immune phenotypes of HLA2DR, CD80 and CD83 for the DCs and of CD3, CD8 and CD56 for the CIK cells, as well as negative detection of bacteria and endotoxin were used as the quality standards of the regimen. The DTH skin test was used to measure the immune response, while physical strength, appetite and sleeping status were analyzed for the subjective clinical outcome. Fever, insomnia, anorexia, joint soreness and skin rashes were recorded as side-effects. Patients received DC vaccination once a week for six weeks and CIK therapy six times within four days.

### Therapy design

The leukocyte fractions were collected on day 0. The DCs were reinfused intravenously on days 8, 15 and 22 and intradermally in the bilateral subaxillary or inguinal region by 24-point injection on days 29, 36 and 43. CIK cells were reinfused intravenously once on days 11 and 13 and twice on days 12 and 14 (Table I).

### Preparation of DCs and CIK cells (17–20)

The patient tumor tissue specimens from the hospital’s tissue bank, or human cell lines corresponding to each cancer type, were mechanically dissociated and a single cell suspension was created for each tumor. After disruption by ultrasound and centrifugation at 600 × g for 30 min, the supernatants were collected as tumor lysate for sensitizing DCs and the DTH test. Patients then underwent leukapheresis with the Fresenius Kabi System. The leukapheresis product, which was enriched for monocytes, was isolated and cultured. Non-adherent cells were cultured in the presence of IFNγ, CD3 monoclonal antibody, IL-2, IL-1 and autologous plasma for 10 days to form CIK cells. Adherent cells were subsequently cultured for seven days with GM-CSF, IL-4, tumor lysate and TNF to form the DC vaccine.

### Quality control of DC vaccine and CIK cells

The prepared DCs and CIK cells were washed. The immune phenotypes of HLA2DR, CD8 and CD83 for the DCs and of CD3, CD8 and CD56 for the CIK cells were analyzed by flow cytometry (FCM). Samples of DCs and CIK cells were cultured for the detection of bacteria, fungus and endotoxin levels. DCs (1×10^7^ cells) were resuspended in 4 ml normal saline (NS) in two syringes for intradermal injection and in 100 ml NS for intravenous injection. The remainder were frozen in 90% autologous serum and 10% dimethyl sulfoxide (DMSO) at 1×10^7^ DCs/ml for further use. The CIK cells (1×10^9^) were resuspended in 100 ml NS for intravenous injection.

### Immune response

The DTH skin test was used as the index of the immune response of the DC vaccine in combination with CIK therapy in the patients with cancer. Tumor lysate (40 *μ*g/0.1 ml) was administrated intradermally into the forearm of each patient one week after the end of therapy. The results of the DTH test were defined as markedly positive, >10 mm diameter of erythema; positive, 5–10 mm; weakly positive, 2–5 mm; and negative, <2 mm after 48 h (Table II).

### Subjective clinical outcome

The improvements in the general condition of the patients, including their physical strength, appetite and sleeping status, were evaluated as the subjective clinical outcomes of the therapy (Table III).

### Safety

Side-effects of fever, insomnia, anorexia, joint soreness and skin rashes were recorded during the process of the therapy (Table IV).

### Statistical analysis

Statistical analyses were performed using the SAS statistical software package (SAS Insititute Inc., Cary, NC, USA). The associations between variables were compared using Pearson’s Chi-square test. P<0.05 was considered to indicate a statistically significant difference.

## Results

### Patient characteristics

A total of 121 cancer patients with histological diagnoses (78 cases) or imaging and medical history diagnoses (43 cases) were included in the present study. The mean age was 60.3 (range, 30–87) years. There were 62 male and 59 female patients. The primary tumors were located in the colorectum (43.0%), lung (15.7%), breast (11.6%), kidney (5.8%), stomach (4.1%) and other regions (19.8%). Among the 121 cases, 72 (59.5%) had tumor loading in their body and 71 (58.7%) received concurrent radiotherapy and/or chemotherapy (Table V).

### Immune response

To test the cell-mediated cytotoxicity response, the DTH skin test was performed at one week subsquent to the end of therapy. Of the 121 patients, 108 underwent the test. Among these 108 cases, 32 were markedly positive, 27 were positive, 24 were weakly positive and 25 were negative 48 h after the DTH skin-test. In total, a 76.9% (83/108) immune response rate was achieved by the therapy (Table II).

### Subjective clinical outcome

To investigate the subjective clinical outcome, improvements in the physical strength, appetite and sleeping status of the patients were recorded in 118 out of 121 cases. Physical strength was recorded in a total of 111 cases (94.1%), with 74 cases of significant and 37 cases of slight improvements in physical strength. Appetite was recorded in a total of 99 cases (83.9%), with 37 cases of significant and 62 cases of slight improvement in appetite. Sleeping status was recorded in a total of 90 cases (76.3%), with 36 cases of significant and 54 cases of slight improvement in sleeping status (Table III).

### Safety

The side-effects were recorded in 118 out of 121 cases. None of the serious adverse effects that are common in chemotherapy and radiotherapy were observed. In total, 69 cases developed a fever during the therapy, which was resolved with antipyretics, dexamethasone and/or physical cooling, 28 cases developed insomnia combined with excitement, 19 cases complained of anorexia, 11 cases complained of joint soreness, which was alleviated with analgesics, and 8 cases developed skin rashes (Table IV).

## Discussion

DCs have been widely used for tumor immunotherapy in several types of cancer. The cytotoxic and regulatory anti-tumor functions of CIK cells have also become attractive targets for immunotherapy. The reciprocal interactions of CIK cells may hold therapeutic promise. The idea of the combined use of CIK cells with DC-based cancer vaccination strategies arises from a number of studies on each strategy (15,21). Targeting the innate and adaptive immune mechanisms may synergistically promote positive clinical outcomes. CIK cells are important in DC-induced antitumor immunity (21–24). DC vaccine regimens in cancer therapy should include evaluations of the CIK cell-stimulating potency. An overview of the effect of the DC vaccine in combination with CIK cell therapy was provided by the present study. In total, 76.9% of patients were positive for the DTH skin-test at 48 h post-treatment. The positive rate of the DTH test in cancer patients receiving the DC vaccine in combination with CIK therapy, as reported in the present study, was significantly higher compared with the use of the DC vaccine therapy alone (83/108 vs. 4/17, P<0.01; Table VI), as reported previously (25).

Although decreases in tumor size observed in MRI or CT scans are often considered to be important assessment indices for cancer therapy, it is difficult to evaluate the clinical outcomes of the regimen and the comprehensive results of the whole anti-cancer therapy. For accurate clinical outcomes, the effect of other combined therapies, such as surgery, radiotherapy or chemotherapy, should be excluded. In order to evaluate the clinical outcomes of the regimen, studies should be performed in cancer patients who are restricted to receiving only the regimen of DC vaccination in combination with CIK therapy. However, this situation only occurs in clinical trails and not in routine clinical therapy, in which multidisciplinary therapy is always used to treat patients with cancer. The present study attempted to use the immune response instead of clinical efficiency to demonstrate the therapeutic effect of the regimen of DC vaccination in combination with CIK therapy (26).

It is generally considered that the detection of the cell surface phenotypes of peripheral blood T lymphocytes by FCM prior to and following DC vaccine therapy is a standard method for evaluating the effect of therapy on the immune function of patients. However, the DTH skin test, with the advantages of being simple to perform, cheap in economy and efficient at indicating an immune response, may be an alternative choice of efficiency index for indicating the clinical response of cancer patients undergoing DC vaccination in combination with CIK therapy (27,28). A positive DTH response indicates that the DC vaccine and CIK cells injected into the body have affected the patients’ immune system, suggesting that the regimen had an immunological function, which should correspond to clinical anti-cancer efficiency. As well as the increase in the immune responses of patients in the present study, the subjective clinical outcomes improved rapidly. In total, 94.1, 83.9 and 76.3% of cases exhibited improvements in physical strength, appetite and sleeping status, respectively.

The combined therapy was safe and could be performed on outpatients. No serious adverse side-effects similar to those caused by chemotherapy and radiotherapy were observed in all 121 cases. During the therapy, 58.5% of cases developed a fever, 23.7% of cases developed insomnia combined with excitement, 16.1% of cases complained of anorexia, 9.3% of cases complained of joint soreness and 6.8% exhibited skin rashes. All these adverse side-effects were well-tolerated and usually alleviated with the relevant treatments.

Patients with advanced cancer usually have a bad general condition due to the development of cancer and repeated use of surgery, radiotherapy or chemotherapy, which may be harmful to the patients in terms of making the treatment ineffective and increasing the susceptibility to tumor progression. The most promising characteristic of the regimen of DC vaccination in combination with CIK treatment in cancer therapy was its low toxicity. Therefore, the regimen was generally well-tolerated with good compliance.

There were no serious adverse side-effects due to the regimen of DC vaccination in combination with CIK cancer therapy. The DC vaccine and CIK cells injected into the body were able to stimulate the patient’s immune systems against the cancer. The regimen may be beneficial to the future treatment of patients with cancer.

## Figures and Tables

**Table I.** Regimen of the DC vaccine in combination with CIK therapy in patients with cancer.

Time	Therapy
Day 0	Collection of leukocyte fractions
Day 8	Reinfusion of DC1 iv
Day 11	Reinfusion of CIK1 iv
Day 12	Reinfusion of CIK2 iv + CIK 3 iv
Day 13	Reinfusion of CIK4 iv
Day 14	Reinfusion of CIK4 iv + CIK5 iv
Day 15	Reinfusion of DC2 iv
Day 22	Reinfusion of DC3 iv
Day 29	Reinfusion of DC4 id
Day 36	Reinfusion of DC5 id
Day 43	Reinfusion of DC6 id

DC, dendritic cell; CIK, cytokine-induced killer.

**Table II.** DTH skin test following the use of the DC vaccine in combination with CIK therapy.

Results of DTH	Definition (mm)	No. (%)
Markedly positive	>10	32 (29.6)
Positive	5–10	27 (25.0)
Weakly positive	2–5	24 (22.2)
Negative	<2	25 (23.1)

The total number of patients was 108 (data loss in 13 out of 121 cases). DTH, delayed-type hypersensitivity; DC, dendritic cell; CIK, cytokine-induced killer.

**Table III.** Subjective clinical outcomes following the use of the DC vaccine in combination with CIK therapy.

Characteristics	Improvement in general condition		

	Significant (%)	Slight (%)	No change or worse (%)
Physical strength	74 (62.7)	37 (31.4)	7 (5.9)
Appetite	37 (31.4)	62 (52.5)	19 (16.1)
Sleeping status	36 (30.5)	54 (45.8)	28 (23.7)

The total number of patients was 118 (data loss in 3 out of 121 cases). DC, dendritic cell; CIK, cytokine-induced killer.

**Table IV.** Side-effects following the use of the DC vaccine in combination with CIK therapy.

Characteristics	No. (%)
Fever	69 (58.5)
Insomnia	28 (23.7)
Anorexia	19 (16.1)
Joint soreness	11 (9.3)
Skin rash	8 (6.8)

The total number of patients was 118 (data loss in 3 out of 121 cases). A patient may have had more than one side-effect. DC, dendritic cell; CIK, cytokine-induced killer. DC, dendritic cell; CIK, cytokine-induced killer.

**Table V.** Patient characteristics.

Characteristic	Value	%
Age (years)		
Range	30–87	
Mean ± SD (range)	60.3±15.9	
Gender, n		
Male	62	51.2
Female	59	48.8
Diagnosis method, n		
Histological diagnosis	78	64.5
Imaging and medical history diagnosis	43	35.5
Tumor type, n		
Colorectal cancer	52	43.0
Lung cancer	19	15.7
Breast cancer	14	11.6
Renal cancer	7	5.8
Gastric cancer	5	4.1
Other	24	19.8
Tumor loading, n		
With	72	59.5
Without	49	40.5
Adjuvant radiotherapy and/or chemotherapy, n		
With	71	58.7
Without	50	41.3

CRC, colorectal cancer.

**Table VI.** Comparison of the results of the DTH test between the DC vaccine and DC vaccine + CIK.

	DTH^+^, n (%)	DTH^−^, n (%)	Total, n	χ^2^	P-value
^*^DC vaccine	4 (23.5)	13 (76.5)	17		
DC vaccine + CIK	83 (76.9)	25 (23.1)	108	19.74	<0.01
Total	87	38	125		

* The data for the DC vaccine therapy alone is cited from our previously reported study (25). DTH, delayed-type hypersensitivity DC, dendritic cell; CIK, cytokine-induced killer.
